# The Treatment of Intra-Peritoneal Injuries

**Published:** 1887-08

**Authors:** 


### THE TREATMENT OF INTRA-PERITONEAL INJURIES.

THIS subject is of especial interest, because of the serious nature and disastrous consequences of wounds of the abdominal walls and viscera, and also on account of the immense progress which has recently been made in the surgical treatment of these injuries. No department of surgery has derived more advantage from that systematic cleanliness which is the basis of our modern system of antiseptics. The old belief in the necessarily fatal character of such injuries, and in the utter futility of operative interference, has given place to a well-grounded hopefulness and a spirit of surgical enterprise, scientifically bold without being rash, which have been the means of saving many a life that would formerly have been condemned.

The expectant treatment which our predecessors adopted, for want of better methods, is no longer considered justifiable, and no surgeon worthy of the name would now allow a case of supposed visceral injury to develop unequivocal symptoms of traumatic peritonitis without doing his best beforehand to ascertain and remedy the injuries which may have been inflicted.

To one who, like Sir William Mac Cormac, has had great experience on the battle-field, such a subject must naturally be congenial. One-tenth of the fatal cases on the field are due to some form of abdominal injury. Although the hurry and excitement of war do not offer a favorable opportunity for successful surgical intervention, still the frequent occurrence of wounds of this kind, and their extremely fatal nature, cannot but excite the interest and attention of those whose lot it is to witness them. The oration delivered by Sir William MacCormac at the annual *conversazione* of the Medical Society on the subject of abdominal section for the treatment of intra-peritoneal injury, gave him an opportunity of reviewing the progress which has been made, and of summing up the knowledge that has been gained, on this important subject. Most surgeons will probably agree with the orator when he expresses the indebtedness of abdominal surgery to Listerism, and the vast improvement both in methods and results which followed its introduction. Thanks to the confidence which experience of its efficacy has engendered, surgeons are now enabled to deal with these cases with a success that would have seemed incredible to surgeons of pre-Listerian times.

Formerly the practice was to suture the abdominal wound, and hope for the best. If the intestines were wounded, death ensued in a large majority of cases. Now, however, if reasonable grounds exist for suspecting intestinal injury, the surgeon at once cuts down and thoroughly explores the abdominal cavity. If no trace of injury be found, he closes the wound and awaits with confidence the issue of the case. He need not reproach himself for having made a useless incision, the danger of which in itself has been reduced to a minimum. If, on the other hand, an incised, lacerated, or perforated wound of the intestines be discovered, instead of abandoning the case he stops the bleeding, fixes the cut edges in close and perfect apposition, and, after the usual cleaning of the peritoneum, replaces everything in position. Numerous cases are now on record proving the triumphs which modern surgery has already achieved, and the future looks yet more promising. By careful observation, confirmed if necessary by experiment, the surgeon learns the manner of dealing with the most sensitive and most easily inflamed tissues of the body. Each little step, each little improvement, helps the movement onwards, until the final result excites the wonder and admiration of those who remember the less daring and less successful practice of a not very remote period.

Since the possibility of succesful intervention in cases of abdominal injury has been demonstrated, the great difficulty to be overcome has lain in the diagnosis. The symptoms of visceral injury, apart from hæmorrhage, are in many cases so obscure, and even so conspicuous by their absence, that the surgeon must often find himself in a very embarrassing position. To operate on an uncertain diagnosis is to expose the patient to an unnecessary risk, while to leave him until the diagnosis can be relied upon seriously lessens the chance of recovery. On the whole, Sir William MacCormac is of opinion that the former course is less dangerous to the patient than inaction.

Not the least interesting portion of the paper is the history of cases in which tentative operations have been performed for severe injuries involving rupture of one or other of the viscera. Though by no means uniformly successful, they mark the path which is to be followed in treating such cases, and point the way to better results in the future. The great point to bear in mind is the desirability of preserving, or, if necessary, restoring, the continuity of the gut. Not only is the patient spared the inconvenience of an artificial anus, but the risks of extravasation are in reality less when the cut edges of the gut are properly sutured than in the alternative proceeding.

The subject of the treatment of wounds of the intestines cannot fail to be of interest to surgeons generally. Even in civil life such cases are frequent enough to render a knowledge of the best methods of treatment indispensable.

Moreover, it is in civil life that the surgeon can interfere with the greatest hope of success. The injuries, as a rule, are less extensive, treatment is more readily available, and the conditions under which it is carried out are incomparably more favorable.—*Quarterly Compendium of Medical Science.*

